# Improving outcomes after pediatric cardiac arrest – the ICU-Resuscitation Project: study protocol for a randomized controlled trial

**DOI:** 10.1186/s13063-018-2590-y

**Published:** 2018-04-03

**Authors:** Ron W. Reeder, Alan Girling, Heather Wolfe, Richard Holubkov, Robert A. Berg, Maryam Y. Naim, Kathleen L. Meert, Bradley Tilford, Joseph A. Carcillo, Melinda Hamilton, Matthew Bochkoris, Mark Hall, Tensing Maa, Andrew R. Yates, Anil Sapru, Robert Kelly, Myke Federman, J. Michael Dean, Patrick S. McQuillen, Deborah Franzon, Murray M. Pollack, Ashley Siems, John Diddle, David L. Wessel, Peter M. Mourani, Carleen Zebuhr, Robert Bishop, Stuart Friess, Candice Burns, Shirley Viteri, David A. Hehir, R. Whitney Coleman, Tammara L. Jenkins, Daniel A. Notterman, Robert F. Tamburro, Robert M. Sutton, for the Eunice Kennedy Shriver National Institute of Child Health and Human Development Collaborative Pediatric Critical Care Research Network (CPCCRN)

**Affiliations:** 1 Department of Pediatrics, University of Utah, 295 Chipeta Way, Salt Lake City, UT 84108 USA; 2 The Learning Centre Institute of Applied Health Research, University of Birmingham, Birmingham, UK; 3 Department of Anesthesiology and Critical Care Medicine, The Children’s Hospital of Philadelphia, University of Pennsylvania, Philadelphia, PA USA; 4 Department of Pediatrics, Children’s Hospital of Michigan, Wayne State University, Detroit, MI USA; 5 Department of Critical Care Medicine, Children’s Hospital of Pittsburgh, University of Pittsburgh, Pittsburgh, PA USA; 6 Department of Pediatrics, Nationwide Children’s Hospital, The Ohio State University, Columbus, OH USA; 7 Department of Pediatrics, UCLA Mattel Children’s Hospital, University of California, Los Angeles, Los Angeles, CA USA; 8 Department of Pediatrics, Benioff Children’s Hospital, University of California, San Francisco, San Francisco, CA USA; 9 Department of Pediatrics, Children’s National Medical Center, George Washington University School of Medicine, Washington, DC USA; 10 Department of Pediatrics, Children’s National Medical Center, Washington, DC USA; 11 Department of Pediatrics, Denver Children’s Hospital, University of Colorado, Denver, CO USA; 12 Department of Pediatrics, Washington University Medical Center, St. Louis, MO USA; 13 Department of Pediatrics, Nemours Alfred I. DuPont Hospital for Children, Wilmington, DE USA; 14 Eunice Kennedy Shriver National Institute of Child Health and Human Development, Bethesda, MD USA; 15 Department of Pediatrics, Milton S. Hershey Medical Center, Pennsylvania State University, Hershey, PA USA

**Keywords:** Cardiopulmonary resuscitation (CPR), Cardiac arrest, Pediatric, In-hospital, Survival, Hybrid, Stepped-wedge

## Abstract

**Background:**

Quality of cardiopulmonary resuscitation (CPR) is associated with survival, but recommended guidelines are often not met, and less than half the children with an in-hospital arrest will survive to discharge. A single-center before-and-after study demonstrated that outcomes may be improved with a novel training program in which all pediatric intensive care unit staff are encouraged to participate in frequent CPR refresher training and regular, structured resuscitation debriefings focused on patient-centric physiology.

**Methods/design:**

This ongoing trial will assess whether a program of structured debriefings and point-of-care bedside practice that emphasizes physiologic resuscitation targets improves the rate of survival to hospital discharge with favorable neurologic outcome in children receiving CPR in the intensive care unit. This study is designed as a hybrid stepped-wedge trial in which two of ten participating hospitals are randomly assigned to enroll in the intervention group and two are assigned to enroll in the control group for the duration of the trial. The remaining six hospitals enroll initially in the control group but will transition to enrolling in the intervention group at randomly assigned staggered times during the enrollment period.

**Discussion:**

To our knowledge, this is the first implementation of a hybrid stepped-wedge design. It was chosen over a traditional stepped-wedge design because the resulting improvement in statistical power reduces the required enrollment by 9 months (14%). However, this design comes with additional challenges, including logistics of implementing an intervention prior to the start of enrollment. Nevertheless, if results from the single-center pilot are confirmed in this trial, it will have a profound effect on CPR training and quality improvement initiatives.

**Trial registration:**

ClinicalTrials.gov, NCT02837497. Registered on July 19, 2016.

**Electronic supplementary material:**

The online version of this article (10.1186/s13063-018-2590-y) contains supplementary material, which is available to authorized users.

## Background

Cardiac arrest occurs in 2–6% of patients admitted to a pediatric intensive care unit (ICU) [1]. Less than half survive to discharge [2–4]. Quality of cardiopulmonary resuscitation (CPR) is associated with survival [5–9], but recommended guidelines are often not met [5, 10]. Although standard courses are the mainstay of ongoing life support training, the evidence that these courses improve outcomes is modest [11–13]. Other approaches to improve CPR quality include real-time CPR feedback devices and structured postresuscitation debriefings. Real-time CPR feedback devices report quantitative CPR quality data (e.g., compression rate, depth, and release velocity) and have been moderately effective at improving psychomotor aspects of basic life support (e.g., chest compressions) [14, 15], but data demonstrating improved long-term outcomes are limited [16]. Comprehensive reviews (debriefings) of resuscitation efforts for the physicians involved in the resuscitation have improved CPR quality but have not been found to increase the rate of survival to hospital discharge [17]. Their lack of effectiveness may be due to traditional debriefing programs targeting only those providers who actually participated in the event under review [17]. However, a novel approach that incorporated patient-centric physiology into the review and included the entire care environment (i.e., both providers who were and those who were not at the resuscitation under review) has been associated with a higher rate of survival with favorable neurologic outcome [18, 19].

The generalizability of the benefits resulting from patient-centric physiologic debriefings with all potential providers of the care environment is unclear because this intervention has been evaluated only in a small, single-center before-and-after study [19]. The 42-month pediatric ICU study included an 18-month control period, a 6-month transition period, and an 18-month intervention period. The study enrolled 52 subjects with an index (first) CPR event in the control period and 42 subjects in the intervention period. The overall rate of survival with favorable neurologic outcome increased from 29% in the control period to 50% in the intervention period. Adjusting for potentially prognostic variables (age, sex, and first documented rhythm), subjects in the intervention period were more likely to survive to hospital discharge with favorable neurologic outcome (adjusted OR 2.75, 95% CI 1.01–7.5; *p* = 0.047) [19]. Despite limitations, the results suggest that this intervention may offer a powerful new approach for improving outcomes of in-hospital pediatric CPR. However, a multicenter randomized controlled trial is needed to assess the generalizability of these findings.

## Methods/design

The present study protocol was written in accordance with Standard Protocol Items: Recommendations for Interventional Trials (SPIRIT). A SPIRIT checklist is provided in Additional file 1.

### Objective

The primary aim of this ongoing multicenter trial, Improving Outcomes from Pediatric Cardiac Arrest – the ICU-Resuscitation Project (ICU-RESUS), is to assess whether structured debriefings for the ICU care environment combined with point-of-care bedside practice will improve the rate of survival with favorable neurologic outcome in children receiving CPR in the ICU.

### Design

**Fig. 1:**
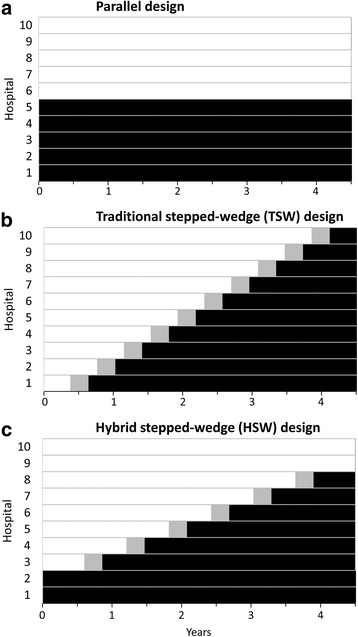
Comparison of cluster-randomized trial designs. This figure illustrates the concept of a hybrid stepped-wedge (HSW) design compared with a parallel group or traditional stepped-wedge (TSW) design. **a** Parallel design. **b** TSW design. **c** HSW design

Three types of designs were considered for this cluster randomized trial: a parallel design, a traditional stepped-wedge (TSW) design [20], and a hybrid stepped-wedge (HSW) design (Fig. 1). In a parallel design, each hospital is randomly assigned to either an intervention or control group for the duration of the trial. In a TSW design, each hospital begins enrolling in the control group and transitions to enrolling in the intervention group at a randomly assigned time. The design ultimately selected for this trial is an HSW design, which is a hybrid between the parallel and stepped-wedge designs. As in a parallel design, two randomly selected hospitals are permanently assigned to enroll in the control group, and two others are permanently assigned to enroll in the intervention group. The remaining six hospitals, as in a stepped-wedge design, initially enroll in the control group but are randomly assigned a time to transition to enrolling in the intervention group. However, it is important to note that the influence of the intervention on hospital staff and thereby on patient outcomes is not fully effective on the day that the intervention is initiated. Therefore, each of these six hospitals has a brief transition period during which outcome data will not be included in the analysis. To our knowledge, ICU-RESUS is the first implementation of an HSW design, one with several advantages in the context of the trial.

An HSW design, like a TSW design, offers a logistically feasible way to roll out a seemingly desirable intervention for participating hospitals at staggered intervals while also allowing a robust comparison between treatment and control periods. In particular, the design enables valid comparisons in the midst of underlying temporal trends in the outcome. In contrast, a simple before-and-after design, as in the single-center pilot study, cannot adequately account for temporal trends, and any observed “intervention effect” cannot be disentangled from other influencing factors that may have changed over time. In addition to the advantages of a TSW design, an HSW design has been reported, in many instances, to have increased power compared with a TSW design or a parallel group design [21].

### Intervention

The intervention is a two-part ongoing training program for ICU staff. The program includes structured debriefings for the entire care environment [19] (i.e., any care provider from several disciplines: physicians, nurses, respiratory therapists, extracorporeal support specialists) and point-of-care practice with a manikin [22].

A 1-h CPR debriefing is held approximately monthly (at least nine times per year) by the unit, and all ICU staff are encouraged to attend. Providers are “invited” to the sessions through emails and departmental flyers. Ideally, a recent resuscitation from within the unit is selected, but a de-identified case from another unit or hospital may be used if needed. The purpose of these debriefings is to carefully review aspects of care that went well and aspects that could be improved [18]. The case introduction includes relevant patient history and details of the patient characteristics and medical management preceding CPR. The debriefing then includes event details such as precode cardiac rhythm, interruptions in compressions, defibrillation attempts with pre- and postshock rhythm, ventilation, compression rate and depth, and release velocity (leaning). When available, case presentations emphasize patient-centric physiologic CPR targets using arterial catheter pressure tracings and end-tidal CO_2_ waveforms. Cases may also include important aspects of pediatric postarrest care such as recognition of seizures [23–25], prevention of fevers [26], and avoidance of hypotension [27]. These sessions are protected under quality improvement guidelines at the individual institutions.

Point-of-care bedside practice allows ICU staff to practice chest compression delivery on a manikin that is set up on a cart so that it can easily be brought to the clinicians throughout the unit. Manikins are equipped to monitor and provide real-time feedback on the quality of delivery (e.g., rate, depth, and release velocity). Both infant and child manikins are available for staff to practice on a model that is relevant to their patients for the shift. Point-of-care practice is frequent (≥ 48 per unit each month), but brief (< 2 minutes) in duration. This component of training was in place during both the control and intervention periods of the single-center pilot study of team debriefings and has been described previously [22, 28–30].

### Participants

ICU-RESUS is supported by the National Heart, Lung, and Blood Institute (NHLBI) and conducted by the *Eunice Kennedy Shriver* National Institute of Child Health and Human Development-funded Collaborative Pediatric Critical Care Research Network (CPCCRN). Hospitals were eligible for participation if they had not implemented either of the elements of the ICU-RESUS intervention and were committed to not implementing the intervention until scheduled by the trial randomization. The ten participating hospitals are geographically diverse tertiary care pediatric hospitals with patients from a mixture of urban, suburban, and rural areas of the United States [31]. These hospitals include 18 distinct units from which subjects will be enrolled.

All eligible subjects at participating institutions are enrolled under a waiver of consent. The central institutional review board at the University of Utah granted a waiver of consent based on the following factors:

1. ICU-RESUS poses no more than minimal risk, because CPR training already exists in the ICUs and resuscitation quality should be at least as good with the ICU-RESUS intervention.
2. The study involves no therapeutic intervention at the patient level, because the intervention is targeted to the care environment and does not deviate from accepted clinical practice.
3. Obtaining informed consent would threaten the scientific validity of the study, which depends on capturing all eligible events during the enrollment period.

Subjects are considered for eligibility if they (1) are ≥ 37 weeks gestational age and ≤ 18 years of age and (2) receive CPR in the ICU. Subjects are excluded if they, prior to the arrest, (1) had a preexisting terminal illness and were not expected to survive the hospitalization (e.g., patients transferred to the ICU for end-of-life care); (2) had a lack of commitment to aggressive ICU therapies; (3) were brain dead; or (4) had an out-of-hospital cardiac arrest associated with the current hospitalization. CPR events lasting < 1 minute will be excluded from analysis if a site investigator determines CPR to have been unnecessary.

**Fig. 2:**
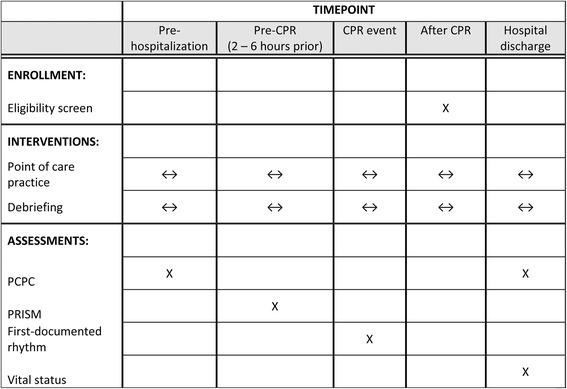
Schedule of enrollment, interventions, and assessments. This figure illustrates the schedule of trial events, including enrollment, interventions, and assessments. Point-of-care practice occurs ≥ 48 times per month in each transitioned or transitioning intensive care unit (ICU). Debriefing occurs at least nine times per year at each transitioned or transitioning ICU. *CPR* Cardiopulmonary resuscitation, *PCPC* Pediatric Cerebral Performance Category scale, *PRISM* Pediatric Risk of Mortality score, a measure of illness severity based on assessments from 2 to 6 h prior to the CPR event

We anticipate enrolling 1540 index CPR events from 18 clinical units at 10 participating hospitals during a 4.5-year enrollment period. A schedule of enrollment, interventions, and assessment is provided in Fig. 2.

### Randomization

The role of randomization in an HSW trial is to determine which clusters will permanently enroll in the control group, which will enroll permanently in the intervention group, and the order in which the others will transition from control to intervention (Fig. 1c). Although the analysis will control for each individual unit, randomization occurs at the hospital level as opposed to the unit level to prevent the possibility of contamination. If units within a hospital were allowed to transition at different times, the intervention in one unit might inadvertently influence outcomes in the other, nontransitioned unit. One way that contamination could happen is through staff members from a transitioned unit temporarily working a shift in the nontransitioned unit. Therefore, when multiple units from a hospital are participating in the trial, all units are assigned the same transition date, reducing the possibility of contamination.

All possible permutations of the hospitals were considered for the order in which hospitals would transition from control to intervention. However, some permutations lead to a transition schedule in which the smallest hospitals transition early and the largest hospitals transition late, causing more subjects to be enrolled in the control group than in the intervention group. The imbalance for each permutation was estimated on the basis of hospital-specific enrollment expectations, and permutations with excessive imbalance were excluded. The permutation selected for implementation was then chosen at random from permutations with adequate balance with respect to expected enrollment between intervention and control groups.

The timing of the intervention for each hospital was established by the data coordinating center prior to enrollment. At a participating hospital randomly selected to transition from control to intervention, investigators and trial staff are informed of their selection 3 months prior to the assigned transition date. This time frame was selected to provide hospitals with sufficient notice to prepare for implementation while not divulging assignment so far in advance as to potentially affect a hospital’s resolve to stay the course. The scheduled hospital is allowed a 4-week window to begin the transition by holding an inaugural debriefing. The exact dates marking the beginning and end of the transition period, a period during which newly enrolled subjects are excluded from analysis, is determined by the date on which the first and fourth debriefings are held. Debriefings are held monthly, making the transition period approximately 3 months.

### Outcome assessment

As in the single center pilot study, the primary outcome is survival to hospital discharge with favorable neurologic outcome. Neurologic outcome is measured with the Pediatric Cerebral Performance Category scale (PCPC [32]) (Table 1) as per standard Utstein-style reporting guidelines [33]. Survivors are considered to have a favorable neurologic outcome if they have no worse than moderate disability at hospital discharge (PCPC score of 1, 2, or 3) or if their PCPC is no worse than at baseline. Baseline PCPC is retrospectively assessed in enrolled patients and is typically based on their status prior to the event leading to the current hospitalization. However, baseline PCPC is evaluated no more than 30 days prior to CPR for subjects who have been in the hospital for more than 90 days. Subjects who are born during the same hospitalization in which the CPR occurred will have their baseline PCPC assessed based on their status prior to the decompensation associated with the CPR event. PCPC assessment is performed by unblinded research coordinators on the basis of detailed objective criteria [32].

**Table 1:** Pediatric Cerebral Performance Category scale

Score	Category	Description
1	Normal	At age-appropriate level; school-age child attend regular school
2	Mild disability	Conscious, alert, able to interact at age-appropriate level; regular school but grades perhaps not age-appropriate, possibility of mild neurologic deficit
3	Moderate disability	Conscious, age-appropriate independent activities of daily living; special education classroom and/or learning deficit present
4	Severe disability	Conscious, dependent on others for daily support because of impaired brain function
5	Coma or vegetative state	Any degree of coma, unaware, even if awake in appearance, without interaction with the environment; no evidence of cortex function; possibility for some reflexive response, spontaneous eye-opening, sleep-wake cycles
6	Brain death/death	Brain death, death

### Sample size calculation

In the single-center pilot, the intervention was associated with an increase in survival with favorable neurologic outcome from 29% to 50% (21 percentage points). We anticipate that we will see improvement to a rate similar to that achieved in the single-center pilot, but preliminary data from another study show that CPCCRN hospitals have a higher average baseline rate of survival with favorable neurologic outcome than the single-center pilot [34]. Therefore, our goal was to select a design and sample size that would provide approximately 80% power (at a significance level of α = 0.05) to detect a more conservative increase from 40% to 51% in survival with favorable neurologic outcome. An important factor for sample size calculation in cluster-randomized trials is the intracluster correlation (ICC), a measure of the similarity in outcomes among patients in the same cluster compared with outcomes among patients in different clusters [35]. The ICC was assumed to be 3% on the basis of pilot data from the multicenter Therapeutic Hypothermia after Pediatric Cardiac Arrest In-Hospital Trial [36].

The power and sample size estimates used for planning this trial were based on the method introduced by Hussey and Hughes [37], though generalized to allow for transition periods and implemented in PASS version 15 software [38]. This method uses a Wald Z-test to evaluate a null hypothesis of no effect against a two-sided alternative. We considered several trial design configurations, including parallel group, TSW, and HSW designs. We also investigated the impact of transition duration. With an ICC of 3%, an HSW design was found to be optimal (Table 2) [21, 39]. Long transition periods in HSW and especially in TSW designs increased the trial duration necessary to achieve > 80% power (Table 2). Therefore, an HSW design was selected with short (3-month) transitions.

**Table 2:** Required duration of enrollment to achieve > 80% power

	Duration of enrollment (years)^a^
Trial design	
Parallel	NA^b^
Traditional stepped-wedge	
6-month transition	6
3-month transition	5.25
Hybrid stepped-wedge	
6-month transition	4.75
3-month transition	4.5

^a^Duration of enrollment is the approximate number of years required to achieve > 80% power to detect an increase from 40% to 51% in the rate of survival with favorable neurologic outcome

^b^In this context, a parallel design cannot achieve 80% power, regardless of the duration of enrollment

To estimate power for the HSW design, we assume that two hospitals, each with two units, are randomly assigned to begin with the intervention, and similarly that four units at two hospitals are assigned to remain as control hospitals for the duration of the study. Enrollment was assumed to be evenly distributed across the 18 units and also across the 18 time periods. A 3-month transition period was included for each of the ten transitioning units in the design-pattern matrix (Table 3). Units are the clusters used for sample size calculations and planned analyses. However, as noted above, randomization occurs at the hospital level to minimize the possibility of intervention contamination between units within a hospital. We found that analyzing 1256 index events (4 per unit per period) would provide 77% power to detect an 11-percentage-point increase in the rate of survival with favorable neurologic outcome from a baseline rate of 40%. Power increases to 84% when analyzing 1570 index events (5 per unit per period). On the basis of concavity of the power curve, analyzing 1391 subjects provides > 80% power. On the basis of historical CPR rates in participating units, we anticipate enrolling 1540 index events over the 4.5-year enrollment period. An estimated 48 index events will occur during the transition periods, allowing analysis of the remaining 1492 events and providing > 80% power.

**Table 3:** Design-pattern matrix for sample size calculations in a 4.5-year hybrid stepped-wedge design with 3-month transitions

Unit	Time period (18 × 3 months)																	
	T1	T2	T3	T4	T5	T6	T7	T8	T9	T10	T11	T12	T13	T14	T15	T16	T17	T18
1	0	0	0	0	0	0	0	0	0	0	0	0	0	0	0	0	0	0
2	0	0	0	0	0	0	0	0	0	0	0	0	0	0	0	0	0	0
3	0	0	0	0	0	0	0	0	0	0	0	0	0	0	0	0	0	0
4	0	0	0	0	0	0	0	0	0	0	0	0	0	0	0	0	0	0
5	0	0	0	0	0	0	0	0	0	0	0	0	0	0	0	.	1	1
6	0	0	0	0	0	0	0	0	0	0	0	0	0	0	0	.	1	1
7	0	0	0	0	0	0	0	0	0	0	0	0	.	1	1	1	1	1
8	0	0	0	0	0	0	0	0	0	0	0	0	.	1	1	1	1	1
9	0	0	0	0	0	0	0	0	0	.	1	1	1	1	1	1	1	1
10	0	0	0	0	0	0	0	0	0	.	1	1	1	1	1	1	1	1
11	0	0	0	0	0	0	.	1	1	1	1	1	1	1	1	1	1	1
12	0	0	0	0	0	0	.	1	1	1	1	1	1	1	1	1	1	1
13	0	0	0	.	1	1	1	1	1	1	1	1	1	1	1	1	1	1
14	0	0	0	.	1	1	1	1	1	1	1	1	1	1	1	1	1	1
15	1	1	1	1	1	1	1	1	1	1	1	1	1	1	1	1	1	1
16	1	1	1	1	1	1	1	1	1	1	1	1	1	1	1	1	1	1
17	1	1	1	1	1	1	1	1	1	1	1	1	1	1	1	1	1	1
18	1	1	1	1	1	1	1	1	1	1	1	1	1	1	1	1	1	1

Each cell represents the intervention status of a unit in a given 3-month period. Zero indicates that the unit has not started the intervention; 1 indicates that the unit has completed the transition; and a dot indicates that the unit is in midtransition

### Data management

Data collected for this trial are entered into OpenClinica, a web-based electronic data capture system, and stored on a secure server at the data coordinating center. An automated system for validating data against a set of predefined rules will query clinical centers regarding data that are invalid, illogical, or incomplete. Data elements critical to the primary aim of this trial are monitored remotely by the data coordinating center to confirm the accuracy of entered data compared with the source documents. Remote monitoring is performed on initial subjects enrolled at each clinical center and then on a subset of additional subjects enrolled throughout the trial.

### Statistical analysis

The primary analysis is to assess the effect of the intervention on survival with favorable neurologic outcome. This analysis is based on subjects enrolled during the control period or the intervention period and excludes subjects enrolled during the transition period. Secondary events (after the first eligible CPR event) during the hospitalization are excluded. The effect of the intervention will be assessed with the following test of hypothesis:

- H_0_: The intervention does not affect the rate of survival with favorable neurologic outcome.
- H_1_: The intervention increases or decreases the rate of survival with favorable neurologic outcome.

Survival with favorable neurologic outcome will be the outcome for a multivariable logistic regression model. The primary predictor in the model will be treatment group (intervention vs. control). Fixed covariates in the model will be age category (≤ 6 months, > 6 months), Pediatric Risk of Mortality score (PRISM; a measure of illness severity in the 2–6 h prior to CPR) [40], first documented rhythm, illness category (medical cardiac, surgical cardiac, medical noncardiac, surgical noncardiac), and a piecewise linear spline of time. Age, illness category and severity, and first documented rhythm are known predictors of mortality and are included to improve the model fit and potentially increase power. In contrast, survival with favorable neurologic outcome is not expected to change over time. However, without adjusting for time, an improvement over time in survival with favorable neurologic outcome that is unrelated to the intervention would be erroneously interpreted as evidence for the intervention owing to the increased enrollment in the intervention group toward the end of the trial. Including time as a covariate is critical for TSW and HSW designs in order to have valid inference in case of potential temporal trends. Although time is often modeled as a categorical variable in TSW trials, we chose to model time as a piecewise linear spline because we expect it to provide better model fit with fewer estimated parameters. For the spline, knots will divide the enrollment period into epochs of equal duration. The number of knots will be chosen to minimize the Akaike information criterion. There are 18 distinct units at the 10 participating hospitals. Unit will be included as a random effect to account for variability in outcomes between units and increase the generalizability of results.

The data needed for this analysis are readily available, and automated data validation ensures that critical measurements are entered. However, in a very small number of subjects, the window for the PRISM score will be entirely prior to hospitalization. In order to obtain a best estimate of prearrest severity of illness, measurements more than 30 minutes prior to the arrest may be used in this case. In the very rare case that a subject experiences a CPR event within the first 30 minutes of hospitalization, all values will be assumed to be normal prior to the decompensation associated with the CPR event.

This trial compares the effect of the ICU-RESUS intervention to current practice, but participating units had varied quality improvement programs prior to this trial. Although participating units are committed to implementing the ICU-RESUS intervention as scheduled by randomization, they are free to continue, modify, or supplement existing programs with other interventions. The programs in place in each unit will be tracked throughout the trial to allow a description of the control group and an exploratory investigation of additional elements of a successful program.

### Data and safety monitoring

The NHLBI appointed an independent data and safety monitoring board (DSMB) to monitor study progress, adherence to the protocol, participant safety, and efficacy. The DSMB has initially planned to meet after the first, second, and third years of enrollment. Formal efficacy monitoring is not planned until after the second year, owing to the sigmoidal growth of the information fraction for TSW and HSW designs and because very early trends may not be representative. In particular, TSW and HSW designs have many more subjects enrolled in the control group than in the intervention group at interim time points. However, efficacy monitoring remains important to allow early stopping and dissemination of results in case of a very large intervention effect, as was observed in the single-center pilot study [19]. Efficacy monitoring will be conducted with the Lan-DeMets approach for flexible alpha spending [41], using conservative O’Brien-Fleming boundaries for early stopping [42].

## Discussion

More than half of the children experiencing an in-hospital cardiac arrest will die prior to hospital discharge [2–4]. Achieving recommended targets for chest compression rate, depth, and limiting interruptions in CPR have all been associated with cardiac arrest outcome [6, 7, 43–47]. However, several studies have demonstrated that even health care providers have difficulty meeting these metrics during real-life resuscitations [10, 48–52]. CPR quality is variable, and standard training courses have demonstrated limited success [5, 10–13]. In short, conventional training methods are failing.

However, there is preliminary evidence suggesting that structured debriefings for the entire care environment may improve outcomes. In particular, a single-center before-and-after pilot study showed an improvement from 29% to 50% in survival with favorable neurologic outcome [19]. These impressive findings have been highlighted by the Institute of Medicine as an exciting approach that should be disseminated to improve outcomes for the > 200,000 adults and children with in-hospital cardiac arrest in the United States each year [53]. Nevertheless, it is important to validate the results of the small, single-center before-and-after study in a larger multicenter trial.

If the results from the single-center pilot study are found to be generalizable, it will have a profound effect on CPR training and quality improvement initiatives, resulting in improved outcomes for children and their families. Assuming roughly 6000 pediatric in-hospital arrests annually [54] and an 11-percentage-point increase in the rate of survival with favorable neurologic outcome, the successful completion of this trial and national dissemination may save the lives of more than 600 children annually.

A TSW design is a relatively new, pragmatic trial design that is now frequently used to assess the effect of an intervention on a community. The HSW design is similar but often provides more power [21, 39], especially when transition periods are essential. To our knowledge, ICU-RESUS is the first implementation of an HSW design. The ICU-RESUS trial uses an HSW design with short transition periods to reduce the sample size, study duration, and cost. The transition period in the single-center pilot was 6 months, with debriefings held every 6–8 weeks. However, we found that these long transition periods had a detrimental effect on power for HSW designs and especially for TSW designs. Using a shorter but more intense transition period (3 months instead of 6 months with debriefings held every 4 weeks) and using an HSW design rather than a TSW design allowed the duration of the trial to be reduced from 6 years to 4.5 years, with accompanying reduction in sample size and cost. The effect of transition duration is so striking because the most influential observations are those immediately prior to and after crossover. Therefore, when a period of transition is included, the observations containing the most statistical information are lost. The effect is somewhat subdued in an HSW design because some hospitals do not cross over and therefore do not experience a transition. Nevertheless, transition periods for both TSW and HSW trials should be as short as possible while still allowing the effect of the intervention to be fully realized. This was achieved in ICU-RESUS by increasing the intensity of the intervention during the transition with more frequent debriefings, which allowed the transition period to be reduced from 6 to 3 months.

Most of the challenges of implementing an HSW trial are the same as for a trial with a TSW design. However, an HSW design introduces a few unique challenges, including the logistics of implementing an intervention prior to the start of enrollment and institutional willingness to participate. In a TSW trial, the intervention is first applied after a period of enrollment. In an HSW design, some hospitals must start the intervention prior to the enrollment period. This can add to the logistical complexity of study startup. When a transition period is required, such as in the ICU-RESUS trial, the start of enrollment may be delayed until the first hospitals are fully transitioned and ready to enroll in the intervention group. If the timeline is inflexible, enrollment may need to begin before the first hospitals have completed the transition to the intervention, reducing statistical power.

Another potential challenge that is unique to an HSW trial is that hospitals may not be willing to participate in a trial in which they might never have the opportunity to apply the seemingly beneficial intervention. However, if true equipoise exists, there should be no preconceived expectation that receiving the intervention will ensure better outcomes. This issue required substantial discussion, but all investigators and sites agreed that the results of the small, single-center before-and-after pilot do not support a generalizable conclusion about the effect of the intervention or justify broad application of this resource-intensive intervention without further study. Therefore, no reservations were expressed by investigators or by the institutional review board in regard to the study design. To assuage potential concerns that the remaining control hospitals may be deprived of this potentially lifesaving program, the investigators determined a priori that support will be provided for the implementation of the intervention, after trial completion, at those remaining control hospitals if it proves to be effective.

In conclusion, the ICU-RESUS trial uses a pragmatic and innovative design to determine whether structured debriefings coupled with point-of-care bedside practice can provide the much-needed improvement in pediatric resuscitation outcomes. This trial has the potential to profoundly impact pediatric in-hospital resuscitation outcomes as well as training and quality improvement initiatives. Additionally, our ongoing experience in the implementation of this trial may serve as an example to others of the practical benefits and challenges of using an HSW design.

## Trial status

The 4.5-year enrollment period began on October 1, 2016, and will conclude on April 1, 2021. This protocol, version 1, was approved on September 7, 2016.

## Additional file

Additional file 1: SPIRIT checklist. (DOC 101 kb)

## Abbreviations

CPCCRN: Collaborative Pediatric Critical Care Research Network
CPR: Cardiopulmonary resuscitation
DSMB: Data and safety monitoring board
HSW: Hybrid stepped-wedge design
ICC: Intracluster correlation
ICU: Intensive care unit
ICU-RESUS: Improving Outcomes from Pediatric Cardiac Arrest – the ICU-Resuscitation Project
NHLBI: National Heart, Lung, and Blood Institute
PCPC: Pediatric Cerebral Performance Category scale
PRISM: Pediatric Risk of Mortality score
SPIRIT: Standard Protocol Items: Recommendations for Interventional Trials
TSW: Traditional stepped-wedge design
